# Progression-Free Survival and Overall Survival of CDK 4/6 Inhibitors Plus Endocrine Therapy in Metastatic Breast Cancer: A Systematic Review and Meta-Analysis

**DOI:** 10.3390/ijms21176400

**Published:** 2020-09-03

**Authors:** Michela Piezzo, Paolo Chiodini, Maria Riemma, Stefania Cocco, Roberta Caputo, Daniela Cianniello, Germira Di Gioia, Vincenzo Di Lauro, Francesca Di Rella, Giuseppina Fusco, Giovanni Iodice, Francesco Nuzzo, Carmen Pacilio, Matilde Pensabene, Michelino De Laurentiis

**Affiliations:** 1 Department of Breast and Thoracic Oncology, Division of Breast Medical Oncology, Istituto Nazionale Tumori IRCCS “Fondazione G. Pascale”, 80131 Naples, Italy; m.piezzo@breastunit.org (M.P.); m.riemma@breastunit.org (M.R.); s.cocco@breastunit.org (S.C.); r.caputo@breastunit.org (R.C.); d.cianniello@breastunit.org (D.C.); germiradigioia@gmail.com (G.D.G.); dilaurovincenzo87@gmail.com (V.D.L.); f.dirella@istitutotumori.na.it (F.D.R.); g.fusco@istitutotumori.na.it (G.F.); g.iodice@istitutotumori.na.it (G.I.); f.nuzzo@istitutotumori.na.it (F.N.); c.pacilio@istitutotumori.na.it (C.P.); m.pensabene@istitutotumori.na.it (M.P.); 2 Department of Public, Clinical and Preventive Medicine, Medical Statistics Unit, University of Campania Luigi Vanvitelli, 80138 Naples, Italy; paolo.chiodini@unicampania.it

**Keywords:** epidemiology, cancer, metastatic breast cancer, hormone therapy, CDK4/6 inhibitors, overall survival, subgroup analysis, hormone receptors, therapies

## Abstract

The introduction of CDK4/6 inhibitors in combination with endocrine therapy (ET) represents the most relevant advance in the management of hormone receptor (HR) positive, HER2-negative metastatic breast cancer over the last few years. This meta-analysis of randomized controlled trials (RCTs) is aimed to better characterize the efficacy of CDK4/6 inhibitors in some relevant subgroups and to test heterogeneity between different compounds with a particular focus on their ability to improve overall survival (OS). Pooled estimates of hazard ratios (HRs) were computed for progression-free survival (PFS), OS, and objective response rate (ORR) analysis in predefined subgroups to better understand treatment effect concerning specific patients’ characteristics. To estimate the absolute benefit in terms of PFS, pooled survival curves were generated by pooling the data of all trials. A total of eight RCTs were included. Adding a CDK4/6 inhibitor to ET is beneficial in terms of PFS, irrespective of the presence or not of visceral metastases, the number of metastatic sites, and the length of the treatment-free interval (TFI). The addition of CDK4/6 inhibitors produces a significant OS improvement, both in aromatase inhibitor (AI)-sensitive (HR 0.75, 95% CI) and AI-resistant patients (HR 0.77, 95% CI [0.67–0.89]). Pooled data from each single drug show that palbociclib remains the only class member not showing a statistically significant HR for OS (HR 0.83, 95% CI [0.68–1.02]).

## 1. Introduction

Breast cancer (BC) is the most common cancer and the second most common cause of cancer-related death in women, with 276,480 new cases estimated in 2020 in USA, about 30% of all diagnosed tumours [1]. Despite the important advances in terms of prevention, diagnosis, and availability of new treatments, metastatic breast cancer (MBC) remains an incurable disease and its evolution depends on several factors, such as site and extension of metastasis, histopathological characteristics, and molecular profiles of tumours.

In the last decade, preclinical and clinical research focused on identification of new treatment options able to prolong or restore endocrine sensitivity, delaying the use of chemotherapy, and improving the survival and the quality of life of these patients [2]. In this scenario, deregulation of the cell cycle represented one of the most interesting therapeutical targets, since dysregulation of the cyclin D-CDK4/6-pRb pathway is frequent in hormonal receptor (HR) positive BC and represents a key mediator of endocrine resistance [3,4,5,6,7,8,9]. Targeting CDK4/6 resulted in an efficient inhibition of this pathway in HR+ BC [10,11]. Despite promising clinical outcomes, intrinsic or acquired resistance to CDK4/6 inhibitors, depending on various mechanisms, can limit the success of these treatments, leading to progression of disease. In this context, knowledge of events leading to disease progression has garnered special interest in breast cancer research. A broad spectrum of events is directly or indirectly responsible for resistance to CDK4/6 inhibitors, including the epithelial–mesenchymal transition (EMT) pathway induced via the activation of TGF-β during the inhibition of the CDK4/6 axis. Several lines of evidence from preclinical and pharmacological studies highlight the role of EMT in maximizing the metastatic potential of breast tumours and its potential inhibition to overcome resistance to CDK4/6 inhibitors [12,13,14,15,16,17,18].

Currently, there are three orally highly selective inhibitors of CDK4/6, namely, palbociclib (PD0332991), ribociclib (LEE011), and abemaciclib (LY2835219).

CDK4/6 inhibitors have been extensively studied in various clinical trials for patients with HR+/HER2-negative MBC. In general, the eligible population in these trials may be classified as follows: (a) sensitive to aromatase inhibitors (AI-sensitive), which includes patients that are either naïve to AI or late relapsers (>12 months) since the stopping of the AI-based adjuvant treatment; or (b) resistant to AI (AI-resistant), which includes patients that are either pretreated with an AI in a metastatic setting or those that relapsed during or early after (≤12 months) the AI-based adjuvant treatment.

Palbociclib was the first inhibitor of CDK4/6 to receive an accelerated approval by the Food and Drug Administration (FDA) in February 2015, in combination with letrozole as initial therapy for AI-sensitive advanced or metastatic BC, based on results from PALOMA-1 and PALOMA-2 trials [19,20,21]. More recently, based on data from PALOMA-3 trial, the indication was expanded to the combination with fulvestrant for both postmenopausal and pre-perimenopausal women with AI-resistant advanced or metastatic BC [22,23,24].

Ribociclib received FDA approval in March 2017, based on results from the MONALEESA-2 trial, as initial endocrine-based therapy for AI-sensitive advanced or metastatic BC, in combination with an aromatase inhibitor and then, based on results from MONALEESA-7 trial, it was also approved for the treatment of peri/premenopausal women [25,26,27,28]. Furthermore, based on the results of the MONALEESA-3 trial [29], ribociclib was also approved in combination with fulvestrant for post-menopausal women with both AI-sensitive and AI-resistant advanced or metastatic BC.

In September 2017, results from the MONARCH-2 trial [30] led to rapid approval of abemaciclib in combination with fulvestrant in women with AI-resistant advanced or metastatic BC. In addition, the MONARCH-3 study showed that the addition of abemaciclib to the non-steroidal aromatase inhibitor (NSAI) significantly improved PFS in patients with AI-sensitive advanced or metastatic BC [31,32]. Abemaciclib has also been approved as a monotherapy for patients with HR-positive/HER2-negative MBC who previously received endocrine therapy (ET) and chemotherapy, based on results from the MONARCH-1 trial [33].

The inclusion of CDK4/6 inhibitors in combination with ET in international treatment guidelines, both for AI-sensitive and AI-resistant patients, represents the most relevant advance in the management of HR-positive/HER2-negative advanced or metastatic BC over the last years [34]. Some controversies, however, still hold, particularly as to whether 1. CDK4/6 inhibitors are effective or necessary for indolent, very late relapsing disease (i.e., those relapsing >38 months from stopping the adjuvant treatment); 2. these drugs are effective in the most aggressive diseases (i.e., early relapse and/or with visceral metastases and/or with a high tumour burden) or where chemotherapy is indicated in these cases; 3. there is different efficacy between different CDK4/6 inhibitors, particularly when the effect on OS is concerned. Indeed, only data from MONALEESA-3, MONALEESA-7 and MONARCH-2 trials show a significant OS improvement with the addition of CDK4/6 inhibitors to ET, while results from PALOMA-1, PALOMA-3 and MONALEESA-2 trials have not shown, so far, a statistically significant increase in OS for the combinations of palbociclib plus letrozole, palbociclib plus fulvestrant, and ribociclib plus letrozole, respectively [24,26,28,35,36,37].

We carried out a metanalysis of all randomized controlled trials (RCTs) with the aims of better characterising the efficacy of CDK4/6 inhibitors in some relevant subgroups of patients and of testing heterogeneity between different compounds with a particular focus on their ability to improve OS.

## 2. Materials and Methods

This systematic review and meta-analysis were conducted using methods proposed by the Cochrane Collaboration, reported in accordance with the PRISMA statement (Supplemental Data), and were registered with PROSPERO (CRD42020161436) [38,39].

### 2.1. Search Strategy

A comprehensive search of MEDLINE via PubMed and the Cochrane databases was performed using medical subject heading (MeSH) terms and text words related to CDK4/6 inhibitors and advanced or metastatic BC. Research was restricted from January 2010 until June 30, 2019, but two additional results, relevant to assess overall survival, were added: MONALEESA-3 and MONARCH-2, presented at the European Society for Medical Oncology Meeting (ESMO) in September 2019. A computerized search was also run in order to identify abstracts and presentations of relevant unpublished studies, reported at the Annual Meetings of the American Society of Clinical Oncology (ASCO), at the San Antonio Breast Cancer Symposium (SABC), and at ESMO meetings over the last three years. Additional studies were hand-searched on Clinicaltrials.gov. Relevant review articles and references from retrieved articles were screened for additional eligible studies. Screening of citations obtained from the literature search was performed by two independent reviewers (MP and PC), as was the assessment of study eligibility. Any disagreement was discussed and resolved by consensus between both reviewers or through consultation with a third reviewer (MDL). A PRISMA flow diagram was prepared to document the process of study selection.

### 2.2. Eligibility Criteria

We included phase II and phase III RTCs which fulfilled the following eligibility criteria: (i) patients with HR-positive/HER2-negative advanced or metastatic BC; (ii) experimental arm including a selective inhibitor of CDK4/6 (palbociclib, ribociclib or abemaciclib); (iii) control arm including standard of care (SOC) treatment ± placebo); (iv) availability of data regarding the primary outcome of interest (PFS/TTP reported in terms of HR and related CIs). SOC treatment included ET only, such as aromatase inhibitors (i.e., anastrozole, letrozole, exemestane), oestrogen receptor modulators (i.e., tamoxifen) or selective oestrogen receptor downregulators (i.e., fulvestrant). Clinical trials that assessed the efficacy of CDK4/6 inhibitor as monotherapy were excluded, as were clinical trials including a chemotherapy-based regimen and studies conducted in a (neo)adjuvant setting. Cohort studies, case series, case reports, and reviews were also excluded.

### 2.3. Outcome of Analysis

The main outcome of interest was the investigator-assessed progression-free survival (PFS), defined as the time from randomisation to objective disease progression or death for any reason. Secondary outcomes were overall survival (OS), defined as the time from randomisation to the date of death due to any cause, and the objective response rate (ORR; proportion of patients achieving a complete or partial response). For PFS and OS analysis, the treatment effect was expressed as the hazard ratio (HR) of the CDK4/6-ET arm over the ET arm, so that an HR greater than one comes out in favour of the standard arm, while an HR less than one comes out in favour of the experimental arm. For ORR, a meta-analysis of single proportions was implemented in order to calculate an overall proportion. All estimates of treatment effect were accompanied by 95% confidence intervals.

### 2.4. Data Extraction and Assessment of Risk of Bias

Data from eligible studies were extracted using a custom-made spreadsheet and were checked for accuracy. The following information were extracted from each study: first author, publication year, ClinicalTrials.gov Identifier, study design, regimen details in both the experimental and control arm, allocated patients for each arm, main patient’s characteristics (median age, line of therapy, sensitivity to ET), and HRs and ORRs for the whole study population and for major subgroups of interest. The 95% CIs related to HRs and ORRs were also extracted. Where available, the full protocol of each study was consulted to verify study objectives, population, and other relevant information regarding study design and conduction. For publications reporting results from the same study, the most recent or complete publication reporting the information of interest was considered.

The risk of bias of included studies was assessed using the Cochrane Collaboration tool; it is made up of six domains: sequence generation, allocation concealment, blinding, incomplete outcome data, selective outcome reporting, and other potential bias [38]. Data extraction and risk of bias for each study were independently assessed by MP and PC, and disagreements were discussed and resolved by consensus between both reviewers or consultation with a third reviewer (MDL).

### 2.5. Statistical Methods

The pooled estimates of HRs and overall proportions of objective responses, with two-sided 95% CIs, were computed for PFS, OS and ORR analysis, by using both a fixed-effect model according the inverse-variance method [40] and the random-effect model of DerSimonian and Laird [41], in order to obtain more appropriate estimates of the average treatment effect in the case of between-study heterogeneity. The assumption of homogeneity between studies was tested with Cochran’s Q statistics [42], and the measure of the degree of inconsistency across studies was assessed with Higgins’ I^2 index [43]. Where available, predefined subgroup analyses were performed, in order to better understand if the treatment effect changed because of specific patient characteristics. Results obtained from the analyses were displayed by generating a forest plot. To estimate the absolute gain in terms of PFS, meta-analytic survival curves were calculated as suggested by Parmar et al. [44]. A sensitivity analysis was carried out by using the leave-one-out cross validation method that recalculates the pooled estimates omitting one study at a time; this analysis is able to capture whether some features of included studies influence the pooled estimates. Publication bias was assessed using funnel plots and regression tests, according to the method reported by Egger [45]. A *p*-value < 0.05 was considered statistically significant. Data analysis was performed using R 3.4.1 software packages [46,47]. To estimate the absolute benefit in terms of PFS, pooled survival curves were generated by pooling the data of all trials, among AI-sensitive and AI-resistant patients. The number at risk and the total number of events were extracted from publications at specific time points based on available data, the survival probabilities were also extracted using DigitizeIt software (I. Bormann, Braunschweig, Germany; https://www.digitizeit.de/).

## 3. Results

### 3.1. Study Selection and Characteristics

The search strategy yielded 685 results from Pubmed, the Cochrane database, conferences, and clinicaltrials.gov. After the initial review of titles, 57 duplicates and another 593 results were discarded. We reviewed 35 abstracts, and 21 references were assessed for eligibility. Finally, we identified eight randomized trials which fulfilled the eligibility criteria. When possible, the latest publication of each trial was used for the meta-analysis. Two additional publications, presented at the ESMO conference in September 2019, were included after database searching for OS analysis. The PRISMA flow diagram and the complete search strategy are available as Supplemental Data.

The eight RCTs included in this systematic review and meta-analysis were published between 2015 and 2019 and randomised a total of 4580 patients, of which 2802 received a CDK4/6 inhibitor (palbociclib, ribociclib or abemaciclib) in association with ET (NSAI, tamoxifene or fulvestrant), and 1778 received standard ET alone or in combination with a placebo. Overall survival results from MONALEESA-3 and MONARCH-2 studies presented after database searching were also included for secondary outcome analysis [35,36]. The main characteristics of each trial included are summarised in Table 1. Palbociclib was tested in combination with letrozole 2.5 mg/day in the PALOMA-1 and PALOMA-2 trials, for AI-sensitive patients, [19,20,21,48] and in combination with fulvestrant 500 mg every 28 days in the PALOMA-3 trial, for AI-resistant patients [24,49,50,51]. Ribociclib was investigated in combination with letrozole 2.5 mg/day in the MONALEESA-2 trial for AI-sensitive post-menopausal women [25,26,28,52,53,54], in combination with tamoxifene or NSAI (with goserelin to suppress ovarian function) in premenopausal AI-sensitive women in the MONALEESA-7 trial [28,55], and in combination with fulvestrant both for AI-sensitive and AI-resistant patients in the MONALEESA-3 trial [30]. Finally, abemaciclib was used in combination with anastrozole 1 mg/day or letrozole 2.5 mg/day, as per physician’s choice, for AI-sensitive patients in the MONARCH-3 trial, [31,32] and in combination with fulvestrant 500 mg every 28 days for AI-resistant patients in the MONARCH-2 trial [30,36]. The main outcome was the PFS for all trials, and OS and ORR were secondary outcomes for all trials. OS estimates were available only for PALOMA-1, PALOMA-3, MONALEESA-2, MONALEESA-3, MONALEESA-7 and MONARCH-2 trials, while OS data were still pending for the PALOMA-2 and MONARCH-3 trials.

### 3.2. Risk of Bias

Overall, the risk of selection, performance, attrition, detection, and reporting bias was very low because all trials were double blind, with the exception of the PALOMA-1 study that was a phase II, open-label study. The risk-of-bias in each study is reported as Supplemental Data.

### 3.3. Progression-Free Survival

PFS hazard ratios were directly available for all included studies. Single study HRs ranged from 0.46 to 0.59 and were all statistically significant. Pooled analysis showed a statistically significant improvement in PFS for patients treated with the CDK4/6 inhibitor in combination with ET versus patients treated with ET alone (HR 0.547 [95% CI 0.504, 0.594], *p*-value < 0.0001). Both a fixed-effect model and a random-effect model were implemented, as initially planned if no heterogeneity between studies was detected (I^2 0%; chi^2 2.95, *p* 0.89) or publication bias (Egger test, *p* 0.09). Forest plots, detection tests for publication bias, and sensitivity analysis are available as Supplemental Data.

### 3.4. Subgroup Analysis

A subgroup analysis was implemented according the following criteria: endocrine sensitivity, site of metastasis, number of organs involved, and treatment-free interval.

#### 3.4.1. AI Sensitivity and Treatment-Free Interval (TFI)

Based on the aforementioned definitions, we pooled PFS estimates for AI-sensitive patients and AI-resistant patients among all trials included in this analysis. Patients with de novo disease were considered a separate group. A total of 5329 patients were included in this group, of which 2852 were AI-sensitive, 1536 were AI-resistant, and 941 patients had de novo disease. MONALEESA-2, MONALEESA-7, MONARCH-3, PALOMA-1, and PALOMA-2 studies enrolled exclusively AI-sensitive patients while MONARCH-2 and PALOMA-3 enrolled AI-resistant patients only; MONALEESA-3 enrolled both sensitive and resistant patients. It also enrolled ‘de novo’ patients (19% of the total), but separate estimates for these patients are not available. ‘De novo’ patients of the MONALEESA-3 trial were therefore included in the AI-sensitive group for the purpose of this meta-analysis. No between-group difference was observed (I^2 0%; chi^2 4.92, *p*-value 0.960). The pooled HRs were very similar in AI-sensitive and ‘de novo’ AI-resistant patients.

**Figure 1:**
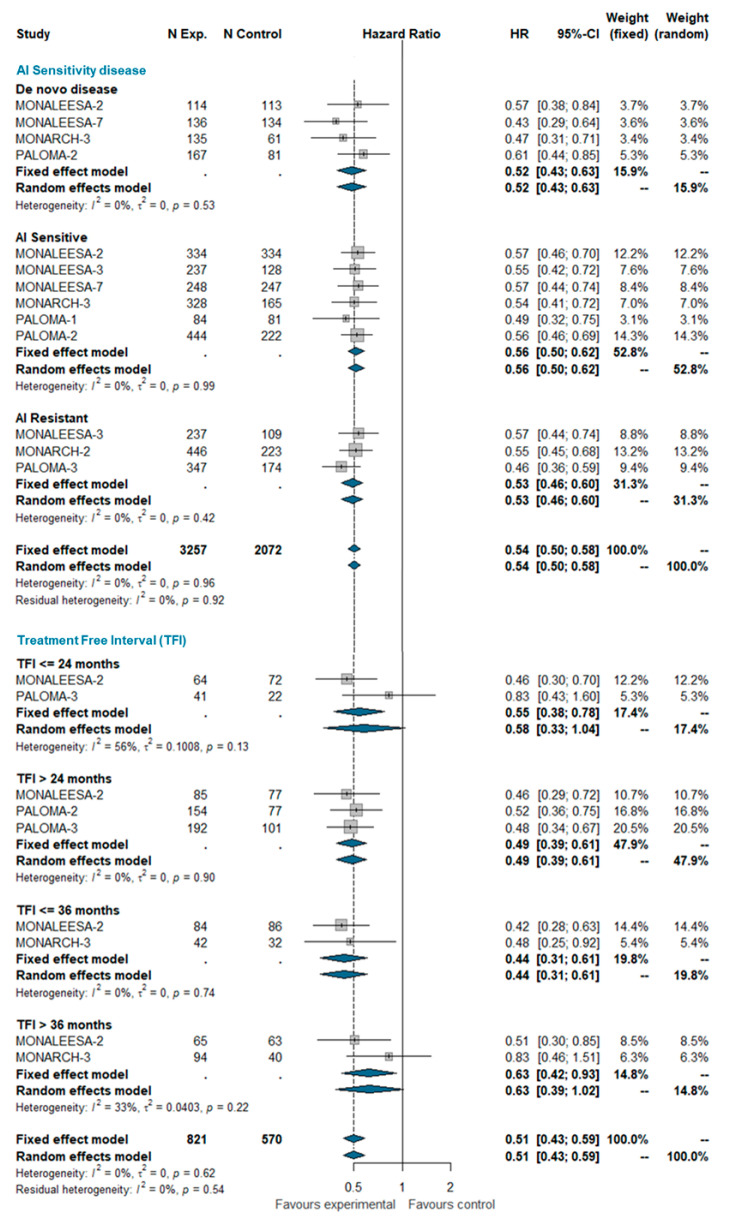
Pooled comparison of PFS according endocrine sensitivity (de novo disease, AI sensitive, AI resistant) and treatment-free interval (TFI <= 24 months, TFI > 24 months, TFI <= 36 months, TFI > 36 months). Abbreviations: PFS: progression free survival; N exp: number of patients randomized in experimental arm; N control: number of patients randomized in control arm; HR: hazard ratio; CI: confidence interval; AI: aromatase inhibitors; TFI: treatment free interval).

The treatment-free interval (TFI) was defined as the time from the end of the adjuvant therapy to randomization. TFI was analyzed at four time points: ≤24 months, >24 months, ≤36 months, and >36 months. Overall, 1391 patients were included in this analysis, of which 199 had a TFI ≤ 24 months, 686 had a TFI > 24 months, 244 had a TFI ≤ 36 months, and 262 had a TFI > 36 months. No significant heterogeneity was observed (I^2 0%; chi^2 6.22, *p*-value 0.622). The pooled analysis confirms the beneficial effect of adding the CDK 4/6 inhibitor to standard ET regardless the treatment-free interval. The estimated pooled HRs according the AI-sensitivity and TFI are shown in Figure 1.

#### 3.4.2. Site of Metastases and Number of Metastatic Sites

**Figure 2:**
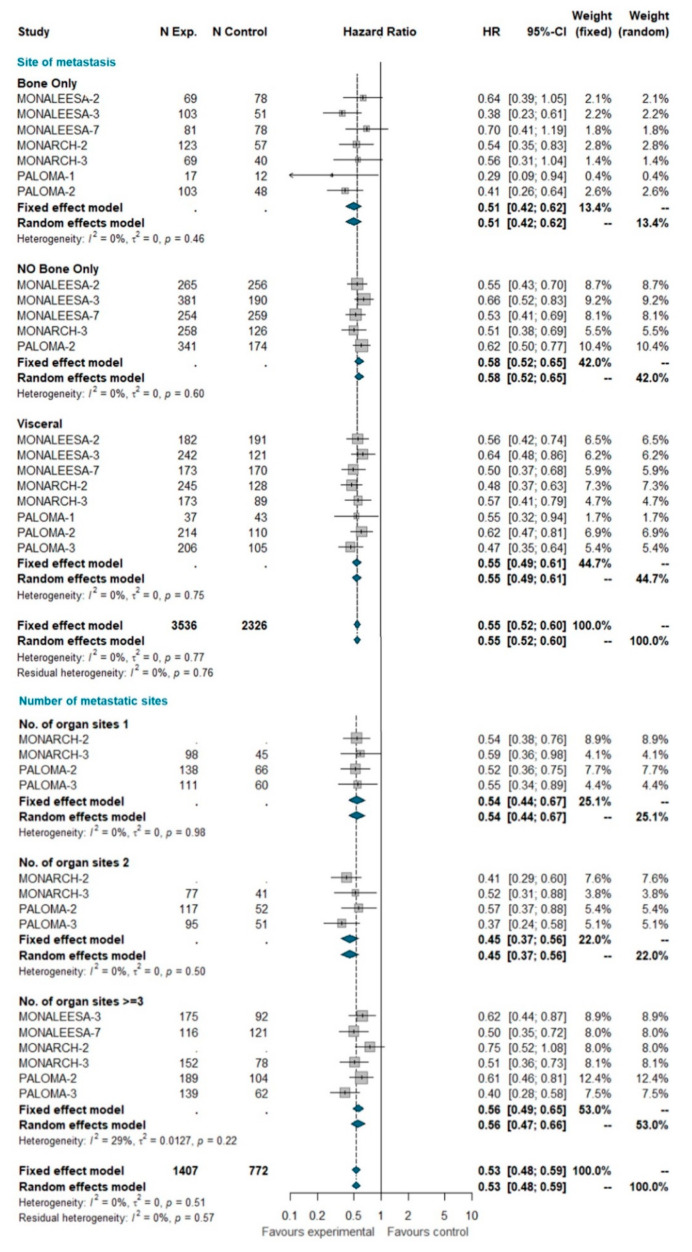
Pooled comparison of PFS according to site of metastasis (visceral disease, bone-only disease and no bone-only disease) and number of organs involved (1, 2, 3+). Abbreviations: PFS: progression free survival; N exp: number of patients randomized in experimental arm; N control: number of patients randomized in control arm; HR: hazard ratio; CI: confidence interval).

A total of 5862 patients were grouped by site of metastasis, of which 2429 had visceral disease (including patients belonging to the liver-or-lung subgroup from MONALEESA-2, MONALEESA-3, and MONALEESA-7 trials), 929 had bone-only disease and 2504 had no bone-only disease (including patients with both visceral and bone disease). The total of 2845 patients was grouped by number of metastatic sites, of which 782 had only one metastatic site at study entry, 635 had two metastatic sites, and 1428 had three or more metastatic sites. No heterogeneity was detected, so the fixed effect model was considered. The pooled results of meta-analysis showed a statistically significant improvement in PFS with a similar HR for all these subgroups (Figure 2).

### 3.5. Objective Response

**Figure 3:**
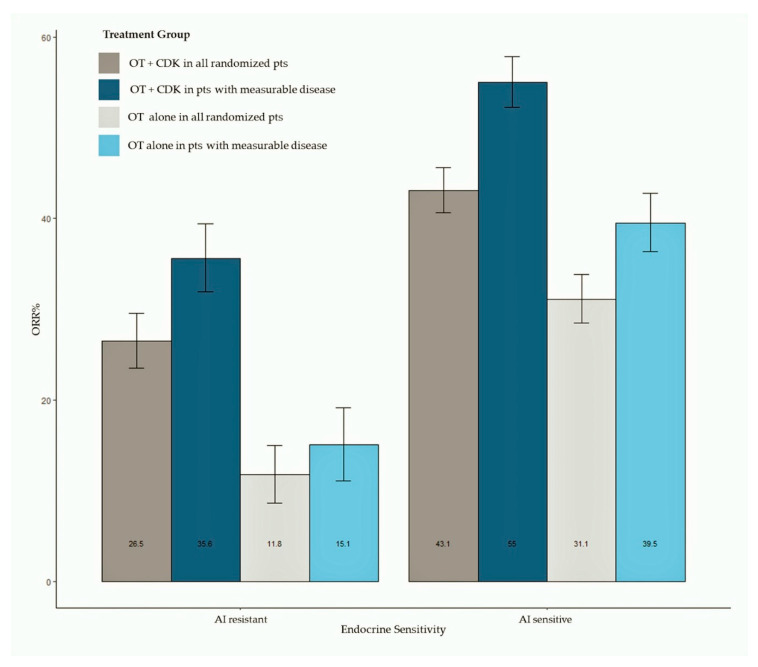
Bar plot of pooled ORR in all randomly assigned patients and in patients with measurable disease according to ET sensitivity status. Abbreviations: ORR: objective response rate; OT: hormonal therapy; CDK: cyclin dependent kinase).

ORR data were available for all studies. Pooled estimates of ORR were summarized as bar plots; forest plots are available as Supplemental Data. Figure 3 shows the bar plot of pooled ORR in all randomly assigned patients and in patients with measurable disease according to AI sensitivity. Overall, 2318 patients treated with the CDK 4/6 inhibitor plus ET and 1536 patients treated with ET alone were included in this analysis, while 1781 patients had measurable disease (1195 treated with the CDK 4/6 inhibitor + ET and 586 treated with ET alone). The meta-analysis shows an increased ORR in patients treated with CDK 4/6 inhibitors, both in AI-sensitive (pooled ORR = 43.3% for CDK4/6 inhibitor-treated patients) and AI-resistant groups (pooled ORR = 26.5% for CDK4/6 inhibitor-treated patients). Patients treated with the CDK 4/6 inhibitor reached a pooled ORR of 55% in the AI-sensitive group and 35.6% in the AI-resistant group. Results of analysis according to the CDK 4/6 inhibitor are available as Supplemental Data.

### 3.6. Overall Survival

**Figure 4:**
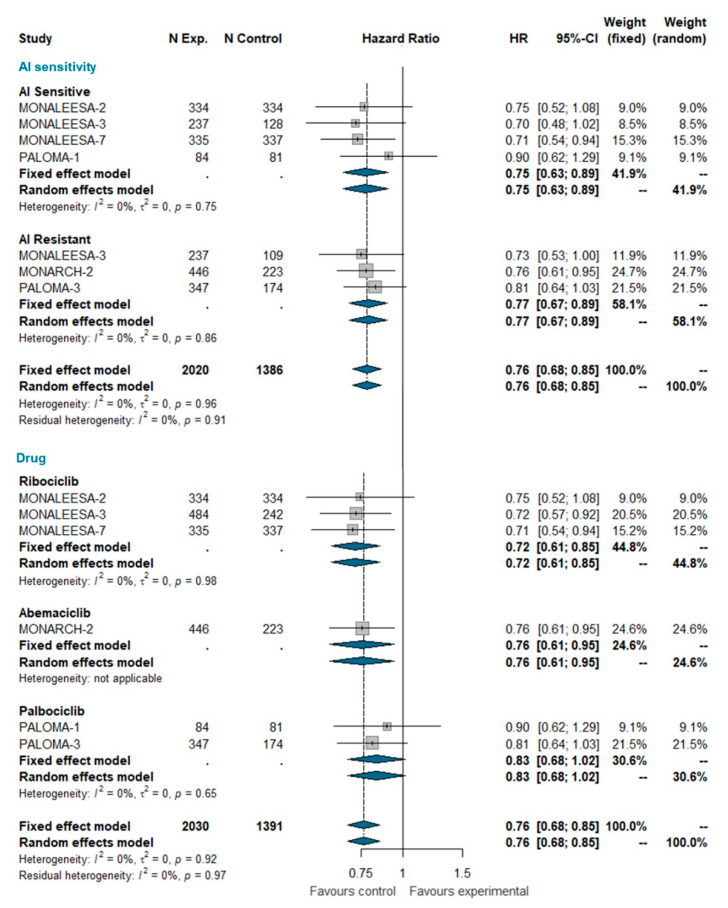
Meta-analysis of overall survival grouped by AI sensitivity (AI sensitive versus AI resistant) and CDK 4/6 inhibitor (Ribociclib, Abemaciclib, Palbociclib). Abbreviations: OS: overall survival; N exp: number of patients randomized in experimental arm; N control: number of patients randomized in control arm; HR: hazard ratio; CI: confidence interval; AI: aromatase inhibitors).

Overall survival (OS) data were available only for MONALEESA-2, MONALEESA-3, MONALEESA-7, MONARCH-2, PALOMA-1, and PALOMA-3 trials [24,27,28,35,36,37]. This analysis included a total of 3421 patients, of which 2030 were treated with CDK 4/6 inhibitors and 1391 were treated with ET alone. The pooled HR indicates a statistically significant reduction in the risk of dying for patients receiving the CDK4/6 inhibitor (HR 0.763 [95% CI 0.683; 0.852], *p*-value < 0.0001); this effect is independent of whether patients were AI sensitive or not. When grouped by CDK4/6 inhibitor, a statically significant reduction in the hazard of dying was apparent for ribociclib and abemaciclib only, but not for palbociclib (Figure 4). However, the test for heterogeneity (I^2 0%; chi^2 1.44, *p*-value 0.919) suggests that discrepant OS results among different CDK4/6 inhibitors may be explained by chance. Meta-analysis of OS in the overall population is available as Supplemental Data.

### 3.7. Pooled Survival Curves

**Figure 5:**
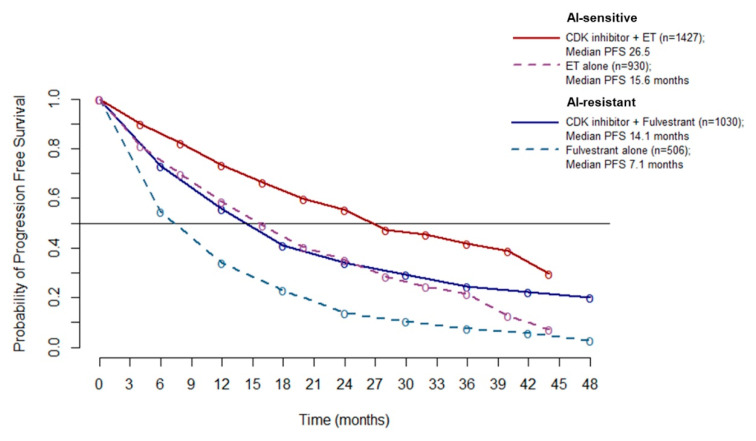
Meta-curves of PFS for AI-sensitive and AI-resistant patients: PFS is significantly longer in patients treated with CDK4/6 inhibitor than patients treated with ET alone (dashed lines), in both AI sensitive (red line and violet dashed line) and AI resistant (blue line and azure dashed line) group. Abbreviations: PFS: progression free survival; AI: aromatase inhibitor; ET: endocrine therapy.

Since no heterogeneity emerged from the overall PFS analysis (A), it is possible to pool the PFS data to better estimate the absolute benefit gained by adding a CDK4/6 inhibitor to ET. Pooled PFS curves show a median PFS of 26.5 months in AI-sensitive patients treated with CDK 4/6 inhibitors (10.9 months improvement over ET alone). In the AI-resistant population, median PFS was 14.1 months for patients treated with CDK 4/6 inhibitors (7 months improvement over ET alone) (Figure 5).

## 4. Discussion

The development of CDK 4/6 inhibitors has changed the therapeutic management of HR+ MBC. Palbociclib, ribociclib, and abemaciclib are all orally active, highly selective reversible inhibitors of CDK4 and CDK6 approved by the Food and Drug Administration, the European Medicine Agency (EMA), and other regulatory agencies worldwide for HR+ MBC in combination with AIs and fulvestrant. While there is general agreement on the efficacy and the manageable toxicity of these drugs, some controversies still hold in the interpretation of clinical trial data, particularly when subgroup data are concerned. This metanalysis was carried out to resolve controversial issues by 1. providing more reliable estimates of efficacy in some controversial subgroups; 2. increasing the statistical power to evaluate the impact on OS; 3. testing for significant heterogeneity between different compounds; 4. refining the overall estimates of efficacy, in the case of no heterogeneity detection.

Despite uniform recommendation from guidelines, chemotherapy is still overused in HR+ MBC, particularly in situations in which an intense tumour debulking is desirable. This behavior relies on the diffused perception, among both oncologists and patients, that chemotherapy is a more potent treatment, yielding on average a higher ORR than ET-based treatments. In contrast, our meta-analysis, as well as the previous ones [56,57,58,59] confirms that the combination of CDK4/6 inhibitors and ET yields a very high rate of tumour regression, which is on average as high as 55% for patients with measurable disease, with no heterogeneity among different compounds. To the best of our knowledge, this ORR is higher than we would expect with mono-chemotherapy and comparable (but still lower) to what we would expect with an aggressive polychemotherapy in HR+ patient populations. Therefore, the use of chemotherapy as the best mean to obtain tumour debulking in HR+ MBC should be definitely considered obsolete. It has been argued that the addition of CDK4/6 inhibitors to ET may not produce relevant benefits for less aggressive tumours, namely, tumours with bone-only metastases or long TFI or limited number of metastases. This debate was based on subgroup analyses from single trials. In contrast, our meta-analysis demonstrates that adding CDK4/6 inhibitors to ET is beneficial in terms of PFS irrespective of the presence of visceral metastases, the number of metastatic sites, and the length of the TFI. The test for heterogeneity in these subgroup analyses indicates that minor differences, if present, are due to chance; we should, therefore, assume that the pooled estimate for the overall population is the best estimate of the treatment effect size in each subgroup of patients.

Overall survival data are relatively immature. Despite this, pooling current estimates from relevant trials demonstrates that the addition of CDK4/6 inhibitors to ET does produce an OS improvement. This improvement is evident both in AI-sensitive patients and AI-resistant patients, strongly supporting the use of CDK4/6 inhibitors as gold standard treatment in both patient populations. A controversial issue is whether these drugs are equally effective in prolonging OS, because, so far, only ribociclib and abemaciclib have demonstrated a statistically significant improvement of OS in at least one trial [60]. In our meta-analysis, when pooling data from different trials for each single drug, palbociclib still remains the only class member to not show a statistically significant HR for OS. However, this result should be interpreted with caution because the test for interaction indicates that the differences between the pooled HRs for the three CDK4/6 inhibitors may well be ascribed to chance (Figure 4). Therefore, as above, one should assume that the best HR estimate for each drug is that of the overall pooled estimate (HR = 0.76; *p* < 0.001) (Figure 4). Yet, because such results pertain to indirect comparisons, we cannot exclude that there could still be moderate, but clinically relevant, differences in efficacy between different compounds, which could only be identified in a direct randomized comparison.

Some limitations of our study need discussion. First, similar to all meta-analyses of published data, publication bias may in theory have overemphasized a positive result. However, we used data from trials published in extenso and from trials reported at meetings to minimize publication bias, and the results of the egger test indicates the absence of a relevant publication bias in our analysis. Second, akin to all studies based on aggregated data, our meta-analysis does not reach the level of evidence obtainable with a meta-analysis based on individual patient data (IPD) because (1) it is impossible to determine the appropriateness of random assignment procedures; (2) trial heterogeneity can only be statistically tested, but never verified; and (3) it is not possible to perform an intention-to-treat analysis because data from excluded patients cannot be retrieved. However, in our case, all authors declared their data were based on the intention-to-treat principle, and the analysis of potential biases indicates that major biases in the included trials are unlikely. Furthermore, provided a rigorous methodology is used, pooling aggregated data, as in our case, yields information that is far superior to the simple description across trial comparison.

## 5. Conclusions

Our meta-analysis confirms the efficacy of CDK4/6 inhibitors overall and in major patient subgroups, highlights differences and similarities between different compounds, and provides pooled (more precise) estimates of the effect size for PFS, OS, and ORR. These results lend further strength to the evidence from single RCTs, supporting the use of CDK4/6 inhibitors in combination with ET as standard treatment for most HR+ MBC patients.

## Figures and Tables

**Table 1:** Characteristics of included studies.

First Author, Year (Study Name)	Phase	Population	Experimental Arm (*n*)	Control Arm (n)	Endocrine Status	Median PFS Exp Arm	Median PFS Ctrl Arm	HR (95% CI)
Hortobagyi GN, 2018 (MONALEESA-2)	III	Post-menopausal AI-sensitive	Ribociclib + Letrozole (334)	Letrozole + Placebo (334)	Sensitive	25.3 (23.0–30.3)	16 (13.4–18.2)	0.568 (0.457–0.704)
Slamon DJ, 2018 (MONALEESA-3)	III	Post-menopausal AI-sensitive/resistant	Ribociclib + Fulvestrant (484)	Fulvestrant + Placebo (242)	Mixed	20.5 (18.5–23.5)	12.8 (10.9–16.3)	0.593 (0.480–0.732)
Tripathy D, 2018 (MONALEESA-7)	III	Pre-menopausal AI-sensitive	Ribociclib + Tamoxifene or NSAI (335)	Placebo + Tamoxifene or NSAI (337)	Mixed	23.8 (19.2–NR *)	13.3 (11.0–16.4)	0.553 (0.441–0.694)
Sledge GW, 2019 (MONARCH-2)	III	Pre/Post-menopausal AI-resistant	Abemaciclib + Fulvestrant (446)	Fulvestrant + Placebo (223)	Resistant	16.4 (not reported)	9.3 (not reported)	0.553 (0.449–0.681)
Johnston S, 2019 (MONARCH-3)	III	Pre/Post-menopausal AI-sensitive	Abemaciclib + NSAI (328)	Placebo + NSAI (165)	Sensitive	28.1 (not reported)	14.7 (not reported)	0.540 (0.418–0.698)
Finn RS, 2015 (PALOMA-1)	II	Pre/Post-menopausal AI-sensitive	Palbociclib + Letrozole (84)	Letrozole (81)	Sensitive	20.2 (13.8–27.5)	10.2 (5.7–12.6)	0.488 (0.319–0.748)
Rugo HS, 2019 (PALOMA-2)	III	Pre/Post-menopausal AI-sensitive	Palbociclib + Letrozole (444)	Letrozole (222)	Sensitive	27.6 (22.4–30.3)	14.5 (12.3–17-1)	0.563 (0.461–0.687)
Cristofanilli M, 2016 (PALOMA-3)	III	Pre/Post-menopausal AI-resistant	Palbociclib + Fulvestrant (521)	Fulvestrant + Placebo (347)	Resistant	9.5 (9.2–11.0)	4.6 (3.5–5.6)	0.46 (0.36–0.59)

* NR, not reached.

## Data Availability

The datasets generated during the current meta-analysis are available from the corresponding author upon reasonable request. All data analysed during this meta-analysis are included in the corresponding published articles, as reported in Table 1.

## Supplementary Materials

The following are available online at https://www.mdpi.com/1422-0067/21/17/6400/s1. Available as separate pdf file.

## Abbreviations

AIs	Aromatase Inhibitors
BC	Breast Cancer
CDK 4/6	Cyclin Dependent Kinase 4/6
CIs	Confidence Intervals
EMA	European Medicine Agency
ET	Endocrine Therapy
FDA	Food and Drug Administration
HR+	Hormone Receptor Positive
IPD	Individual Patient data
MBC	Metastatic Breast Cancer
NSAI	Non Steroidal Aromatase Inhibitors
ORR	Objective Response Rate
OS	Overall Survival
PFS	Progression-Free Survival
SOC	Standard Of Care
TFI	Treatment-Free Interval
TTP	Time To Progression
