# Supplementary material for: Progression-Free Survival and Overall Survival of CDK 4/6 Inhibitors Plus Endocrine Therapy in Metastatic Breast Cancer: A Systematic Review and Meta-Analysis
Source: Int J Mol Sci. 2020 Sep 3;21(17):6400. doi: 10.3390/ijms21176400 (PMC7503849; doi:10.3390/ijms21176400)
Supplement: Supplementary file 1 [file ijms-21-06400-s001.pdf]

M. Piezzo, P. Chiodini, M. Riemma, S. Cocco, R. Caputo, D. Cianniello, V. Di Lauro, F. Nuzzo, M. Pensabene and M. De Laurentiis***: Progression free survival and overall survival of CDK 4/6 inhibitors plus endocrine therapy in metastatic breast cancer: a systematic review and meta-analysis**

Supplemental Data (online only)

*Correspondig author: m.delaurentiis@breastunit.org

Contents:

Figure S1: PRISMA diagram 2

Table S1: Full search strategy 3

Figure S2. Risk of bias for selected studies: review authors' judgements about each risk of bias item for each included study 4

Figure S3. Begg's funnel plot and Egger's test for publication bias detection 5

Table S2. Sensitivity analysis 6

Figure S4. Meta-analysis of progression free survival (PFS) in overall population 7

Figure S5. Meta-analysis of progression free survival (PFS) in peri/premenopausal and postmenopausal patients 8

Figure S6. Meta-analysis of objective response rate (ORR) in patients treated with CDK 4/6 inhibitor plus endocrine therapy according AI-sensititvity 9

Figure S7. Meta-analysis of objective response rate (ORR) in patients treated with endocrine therapy alone according AI-sensititvity 10

Figure S8. Bar-plot of pooled ORR in all randomly assigned patients according CDK 4/6 inhibitor 11

Figure S9. Meta-analysis of overall survival (OS) in overall population 12

Table S3. PRISMA 2009 checklist 13

Figure S1: PRISMA diagram

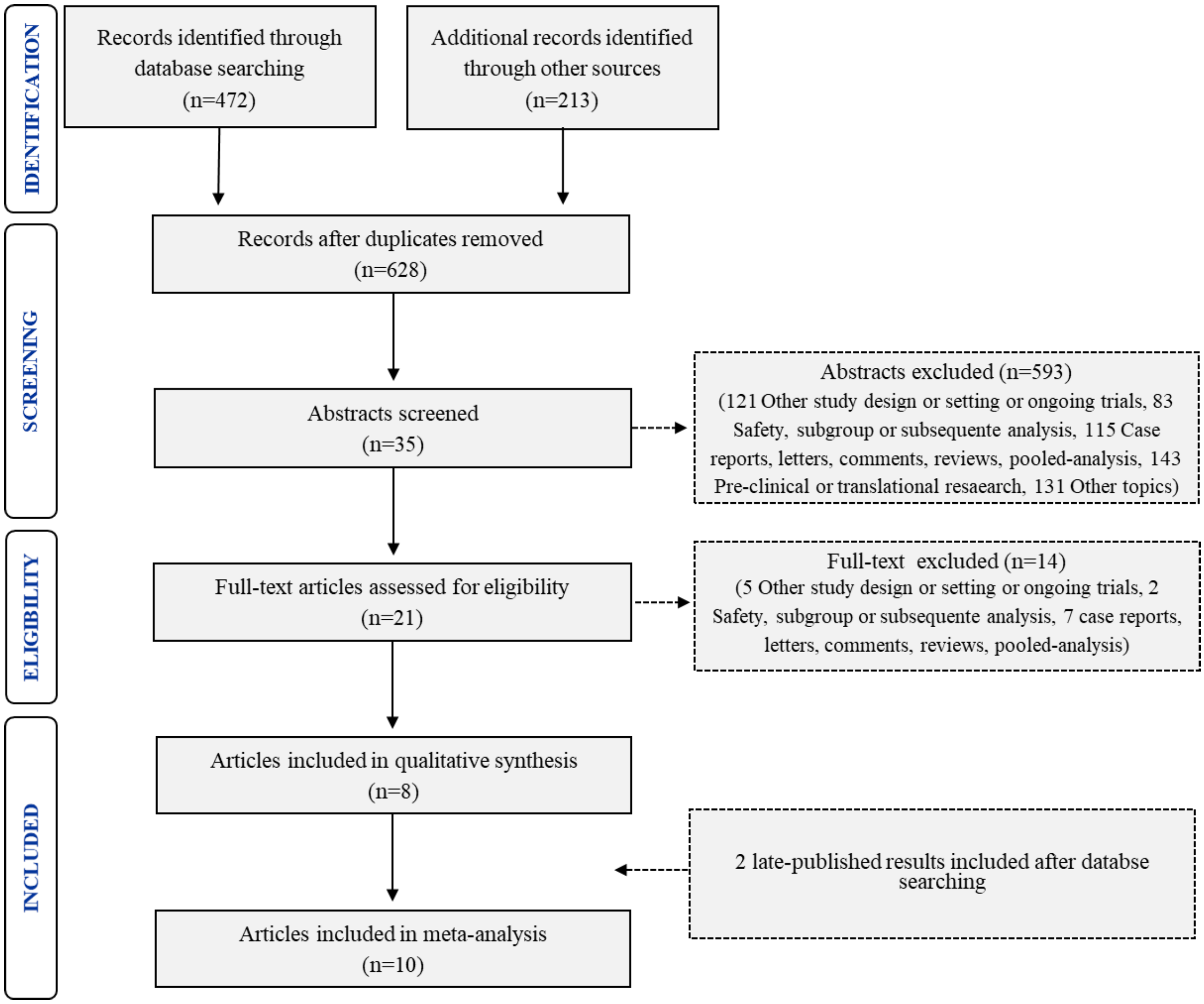

Table S1: Full search strategy

Search ID	Search Details
#1	(advanced[All Fields] OR ("secondary"[Subheading] OR "secondary"[All Fields] OR "metastatic"[All Fields])) AND ("breast neoplasms"[MeSH Terms] OR ("breast"[All Fields] AND "neoplasms"[All Fields]) OR "breast neoplasms"[All Fields] OR ("breast"[All Fields] AND "cancer"[All Fields]) OR "breast cancer"[All Fields])
#2	((((((((((((((((((((cdk[All Fields] AND inhibitor[All Fields]) OR (cdk[All Fields] AND 4/6[All Fields])) OR (cdk4[All Fields] AND 6[All Fields])) OR (cdk[All Fields] AND 4[All Fields] AND 6[All Fields])) OR cdk4/6[All Fields]) OR ("5-(4-ethylpiperazin-1-ylmethyl)pyridin-2-yl)-(5-fluoro-4-(7-fluoro-3-isopropyl-2-methyl-3H-benzimidazol-5-yl)pyrimidin-2-yl)amine"[Supplementary Concept] OR "5-(4-ethylpiperazin-1-ylmethyl)pyridin-2-yl)-(5-fluoro-4-(7-fluoro-3-isopropyl-2-methyl-3H-benzimidazol-5-yl)pyrimidin-2-yl)amine"[All Fields] OR "ly2835219"[All Fields])) OR ("5-(4-ethylpiperazin-1-ylmethyl)pyridin-2-yl)-(5-fluoro-4-(7-fluoro-3-isopropyl-2-methyl-3H-benzimidazol-5-yl)pyrimidin-2-yl)amine"[Supplementary Concept] OR "5-(4-ethylpiperazin-1-ylmethyl)pyridin-2-yl)-(5-fluoro-4-(7-fluoro-3-isopropyl-2-methyl-3H-benzimidazol-5-yl)pyrimidin-2-yl)amine"[All Fields] OR "abemaciclib"[All Fields])) OR verzenio[All Fields]) OR ("palbociclib"[Supplementary Concept] OR "palbociclib"[All Fields] OR "pd 0332991"[All Fields])) OR ("palbociclib"[Supplementary Concept] OR "palbociclib"[All Fields] OR "pd 0332991"[All Fields])) OR ("palbociclib"[Supplementary Concept] OR "palbociclib"[All Fields] OR "pd0332991"[All Fields])) OR ("palbociclib"[Supplementary Concept] OR "palbociclib"[All Fields])) OR ("palbociclib"[Supplementary Concept] OR "palbociclib"[All Fields] OR "ibrance"[All Fields])) OR ("ribociclib"[Supplementary Concept] OR "ribociclib"[All Fields] OR "lee011"[All Fields])) OR ("ribociclib"[Supplementary Concept] OR "ribociclib"[All Fields])) OR kisqali[All Fields]) OR (("cyclin-dependent kinase 4"[MeSH Terms] OR "cyclin-dependent kinase 4"[All Fields] OR "cyclin dependent kinase 4"[All Fields]) AND 6[All Fields])) OR (("cyclin-dependent kinase 4"[MeSH Terms] OR "cyclin-dependent kinase 4"[All Fields] OR "cyclin dependent kinase 4"[All Fields]) AND 6[All Fields])) OR ("cyclin-dependent kinase inhibitor proteins"[MeSH Terms] OR ("cyclin-dependent"[All Fields] AND "kinase"[All Fields] AND "inhibitor"[All Fields] AND "proteins"[All Fields]) OR "cyclin-dependent kinase inhibitor proteins"[All Fields] OR ("cyclin"[All Fields] AND "dependent"[All Fields] AND "kinase"[All Fields] AND "inhibitor"[All Fields]) OR "cyclin dependent kinase inhibitor"[All Fields])) OR (("cyclin-dependent kinase 4"[MeSH Terms] OR "cyclin-dependent kinase 4"[All Fields] OR "cyclin dependent kinase 4"[All Fields]) AND 6[All Fields])) OR (("cyclin-dependent kinase 4"[MeSH Terms] OR "cyclin-dependent kinase 4"[All Fields] OR "cyclin dependent kinase 4"[All Fields]) AND 6[All Fields])
#3	#1 AND #2
#4	#3 AND ("2010/01/01"[Date - Publication] : "2019/06/30"[Date - Publication])
#5	#4 NOT "review"[Publication Type]

Figure S2. Risk of bias for selected studies: review authors' judgements about each risk of bias item for each included study

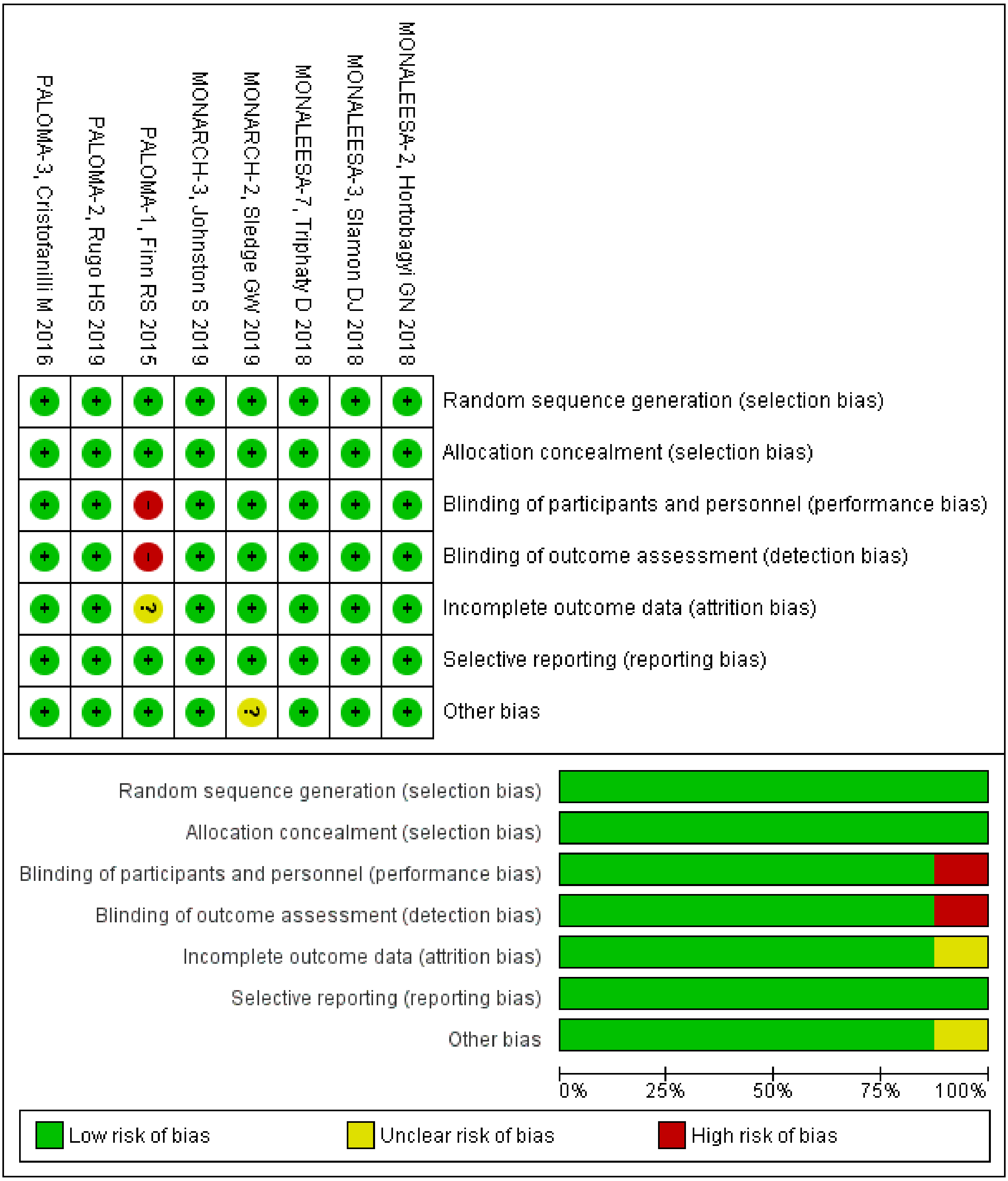

Figure S3. Begg's funnel plot and Egger's test for publication bias detection

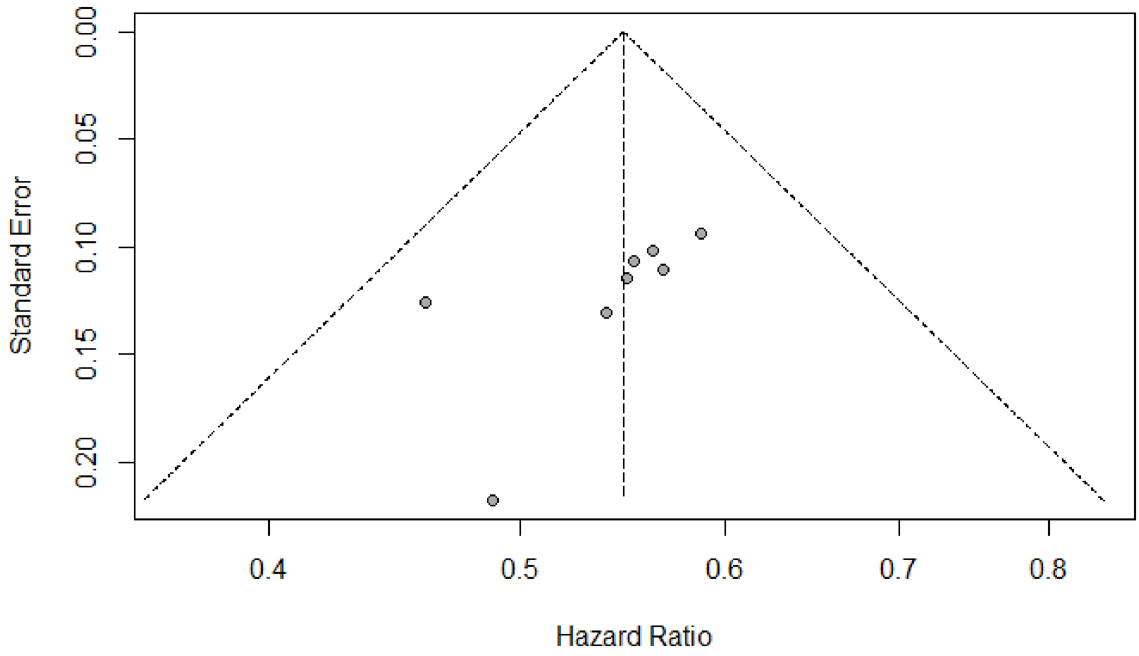

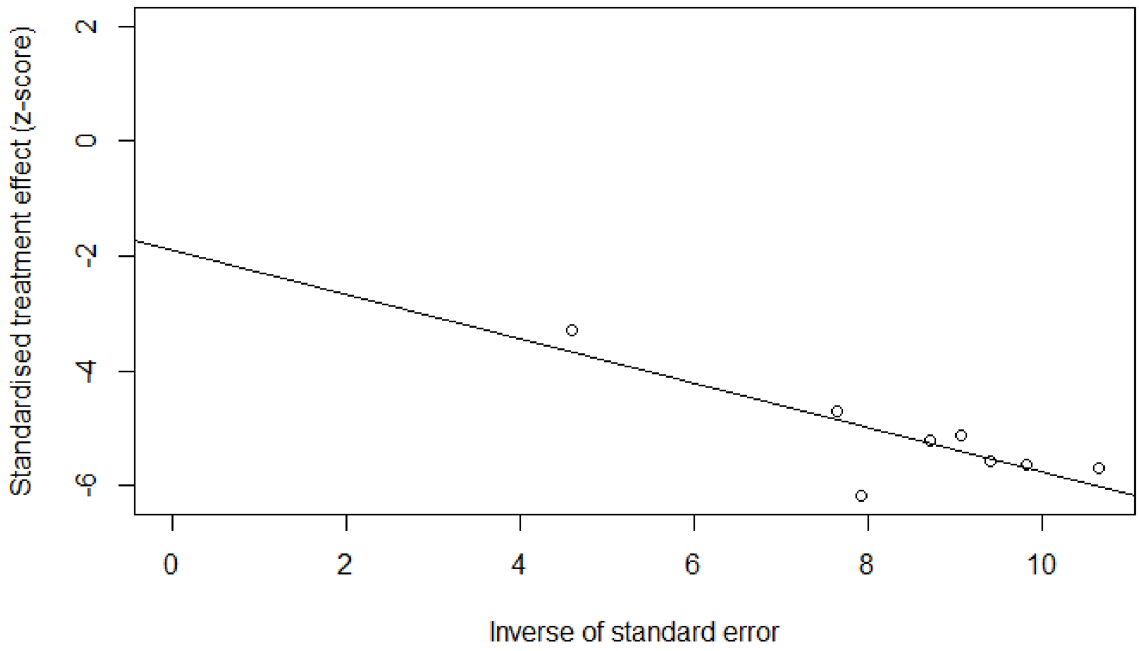

Table S2. Sensitivity analysis

Trial	HR	Zval	Pval	CI low 95%	CI upp 95%	Q	Qp
**Hortobagyi GN, 2018 (MONALEESA-2)**	**0.539**	**-4.732**	**0.000**	**0.418**	**0.697**	**0.351**	**0.999**
**Slamon DJ, 2018 (MONALEESA-3)**	**0.535**	**-4.725**	**0.000**	**0.413**	**0.694**	**0.296**	**1.000**
**Tripathy D, 2018 (MONALEESA-7)**	**0,542**	**-4.707**	**0.000**	**0.420**	**0.700**	**0.370**	**0.999**
**Sledge GW, 2017 (MONARCH-2)**	**0,542**	**-4.678**	**0.000**	**0.419**	**0.700**	**0.680**	**0.999**
**Johnston S, 2019 (MONARCH-3)**	**0,544**	**-4.729**	**0.000**	**0.422**	**0.700**	**0.371**	**0.999**
**Finn RS, 2015 (PALOMA-1)**	**0,547**	**-4.792**	**0.000**	**0.428**	**0.700**	**0.315**	**0.999**
**Rugo HS, 2019 (PALOMA-2)**	**0,540**	**-4.695**	**0.000**	**0.417**	**0.698**	**0.570**	**0.999**
**Cristofanilli M, 2016 (PALOMA-3)**	**0,555**	**-4.553**	**0.000**	**0.431**	**0.715**	**0.123**	**1.000**

Abbreviations: HR, hazard ratio; Zval, value of z-statistic; Pval, p-value related to z statistic; CI low 95%, confidence interval lower limit at 95%; CI upp 95%, confidence interval upper limit at 95%; Q, Q-statistic; Qp, p-value related to Q statistic.

The sensitivity analysis was performed using the leave-one-out method and shows that the estimated pooled HRs, obtained excluding one study at time, are still consistent.

Figure S4. Meta-analysis of progression free survival (PFS) in overall population

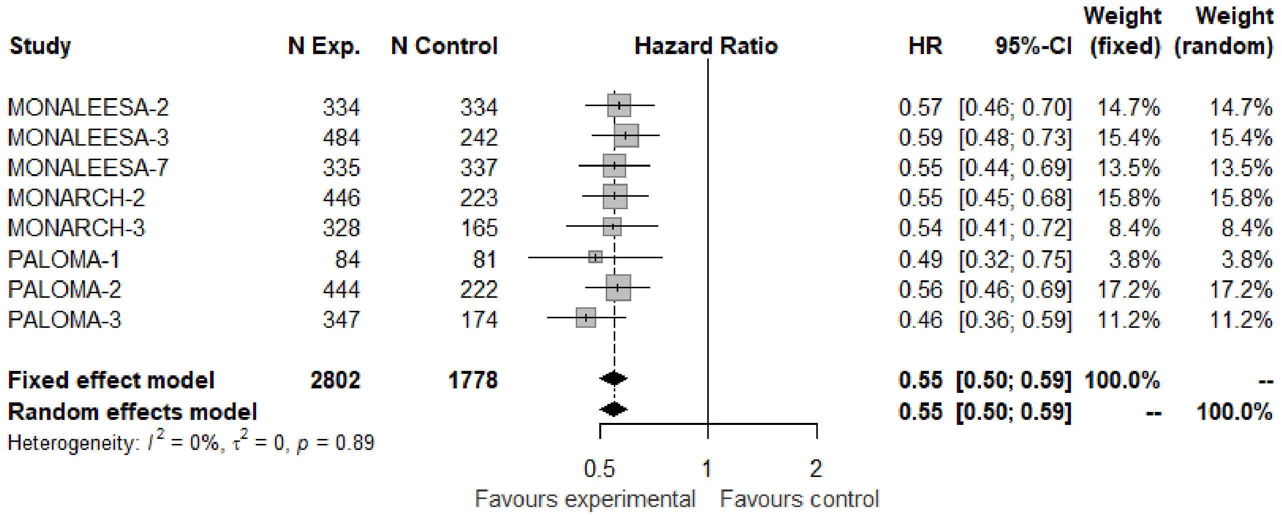

Abbreviations: N exp, number of patients randomized in experimental arm (CDK 4/6 inh + ET); N control, number of patients randomized in control arm (Placebo + ET or ET alone); HR, hazard ratio; 95%-CI, confidence intervals at 95%; Weight (fixed), weight of each study in a fixed effect model; Weight (random), weight of each study in a random effect model.

Studies are ordered by alphabetical order and by year of reporting; squares on the hazard ratio plot are proportional to the weight of each study; weighting is based on the inverse variance method.

Figure S5. Meta-analysis of progression free survival (PFS) in peri/premenopausal and postmenopausal patients

Study	N Exp.	N Control	HR	95%-CI	Weight (fixed)	Weight (random)
**Post-menopausal**						
MONALEESA-2	334	334	0.57	[0.46; 0.70]	13.0%	13.0%
MONALEESA-3	484	242	0.59	[0.48; 0.73]	13.6%	13.6%
MONARCH-2	371	180	0.58	[0.46; 0.73]	12.0%	12.0%
MONARCH-3	328	165	0.54	[0.42; 0.70]	9.2%	9.2%
MONARCH-3	328	165	0.54	[0.42; 0.70]	9.2%	9.2%
PALOMA-1	84	81	0.49	[0.32; 0.75]	3.3%	3.3%
PALOMA-2	444	222	0.56	[0.46; 0.69]	15.3%	15.3%
PALOMA-3	275	138	0.45	[0.34; 0.59]	8.0%	8.0%
**Fixed effect model**	**2648**	**1527**	**0.55**	**[0.51; 0.60]**	**83.8%**	**--**
**Random effects model**			**0.55**	**[0.51; 0.60]**	**--**	**83.8%**
Heterogeneity: $I^2 = 0\%$, $\tau^2 = 0$, $p = 0.86$						
**Pre/Peri-menopausal**						
MONALEESA-7	335	337	0.55	[0.44; 0.69]	12.0%	12.0%
MONARCH-2	72	42	0.42	[0.25; 0.70]	2.2%	2.2%
PALOMA-3	72	36	0.50	[0.29; 0.87]	2.0%	2.0%
**Fixed effect model**	**479**	**415**	**0.52**	**[0.43; 0.63]**	**16.2%**	**--**
**Random effects model**			**0.52**	**[0.43; 0.63]**	**--**	**16.2%**
Heterogeneity: $I^2 = 0\%$, $\tau^2 = 0$, $p = 0.61$						
**Fixed effect model**	**3127**	**1942**	**0.55**	**[0.51; 0.59]**	**100.0%**	**--**
**Random effects model**			**0.55**	**[0.51; 0.59]**	**--**	**100.0%**
Heterogeneity: $I^2 = 0\%$, $\tau^2 = 0$, $p = 0.93$						
Residual heterogeneity: $I^2 = 0\%$, $p = 0.90$						

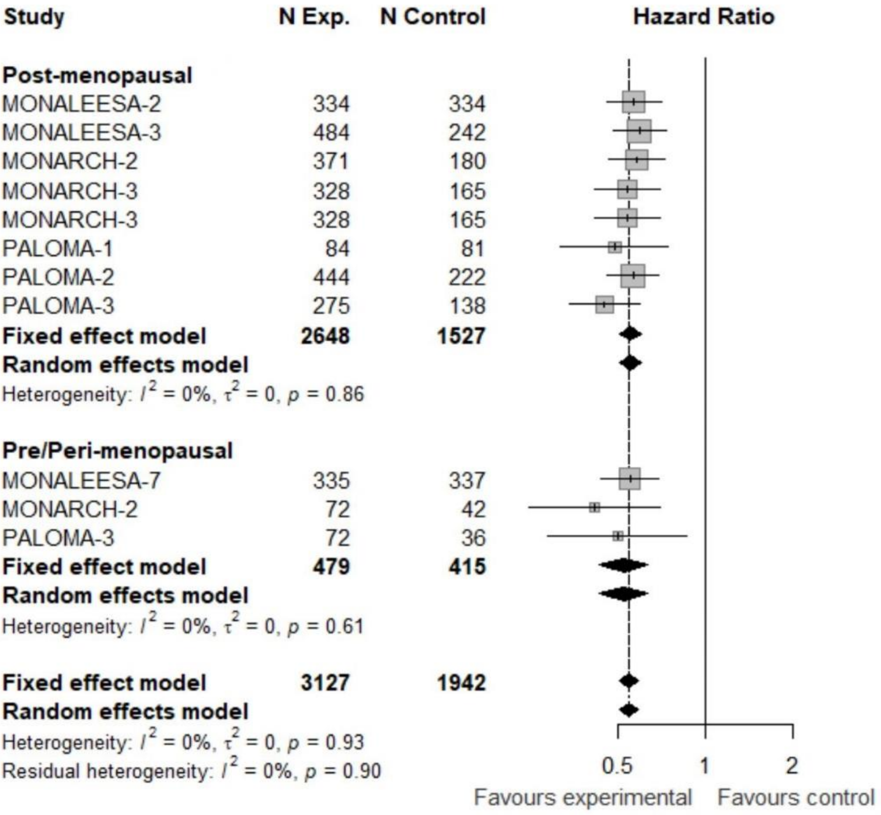

Abbreviations: N exp, number of patients randomized in experimental arm (CDK 4/6 inh + ET); N control, number of patients randomized in control arm (Placebo + ET or ET alone); HR, hazard ratio; 95%-CI, confidence intervals at 95%; Weight (fixed), weight of each study in a fixed effect model; Weight (random), weight of each study in a random effect model.

Studies are ordered by alphabetical order and by year of reporting; squares on the hazard ratio plot are proportional to the weight of each study; weighting is based on the inverse variance method.

Figure S6. Meta-analysis of objective response rate (ORR) in patients treated with CDK 4/6 inhibitor plus endocrine therapy according AI-sensititvity

Study	OR	Total	ORR (%)	95% CI	Weight
**ET Status = Sensitive**					
MONALEESA-2	136	334	40.7	[35.4; 46.2]	14.5%
MONALEESA-7	137	335	40.9	[35.6; 46.4]	14.5%
MONARCH-3	163	328	49.7	[44.2; 55.2]	14.4%
PALOMA-1	36	84	42.9	[32.1; 54.1]	12.2%
PALOMA-2	186	444	41.9	[37.3; 46.6]	14.7%
**Fixed effect model**		**1525**	**43.1**	**[40.6; 45.6]**	**--**
**Random effects model**			**43.2**	**[39.7; 46.7]**	**70.4%**
Heterogeneity: $I^2 = 46\%$, $\tau^2 = 0.0007$, $p = 0.11$					
**ET Status = Resistant**					
MONARCH-2	157	446	35.2	[30.8; 39.8]	14.8%
PALOMA-3	66	347	19.0	[15.0; 23.6]	14.8%
**Fixed effect model**		**793**	**26.5**	**[23.5; 29.6]**	**--**
**Random effects model**			**27.1**	**[11.2; 42.9]**	**29.6%**
Heterogeneity: $I^2 = 96\%$, $\tau^2 = 0.0126$, $p < 0.01$					
**Fixed effect model**		**2318**	**36.4**	**[34.5; 38.4]**	**--**
**Random effects model**			**38.4**	**[30.3; 46.6]**	**100.0%**
Heterogeneity: $I^2 = 94\%$, $\tau^2 = 0.0112$, $p < 0.01$					
Residual heterogeneity: $I^2 = 86\%$, $p < 0.01$					

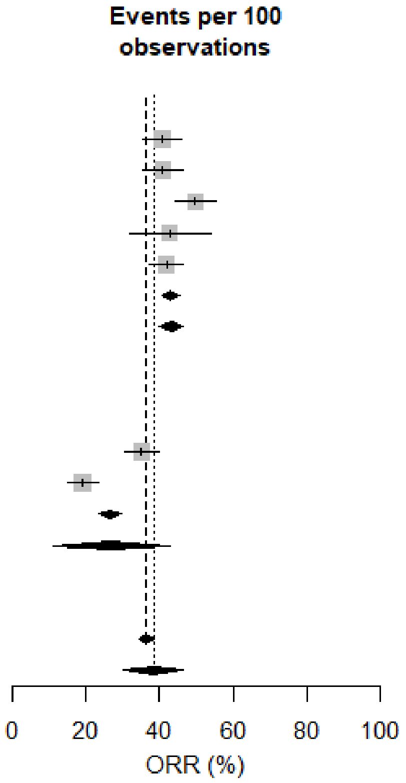

A meta-analysis of single proportions was carried out to obtain the pooled estimate of ORR in patients treated with CDK 4/6 inhibitor plus endocrine therapy according their AI-sensititvity.

Figure S7. Meta-analysis of objective response rate (ORR) in patients treated with endocrine therapy alone according AI-sensititvity

Study	OR	Total	ORR (%)	95% CI	Weight
**ET Status = Sensitive**					
MONALEESA-2	92	334	27.5	[22.8; 32.7]	14.8%
MONALEESA-7	100	337	29.7	[24.8; 34.9]	14.8%
MONARCH-3	61	165	37.0	[29.6; 44.8]	13.9%
PALOMA-1	27	81	33.3	[23.2; 44.7]	12.6%
PALOMA-2	77	222	34.7	[28.4; 41.3]	14.3%
**Fixed effect model**		**1139**	**31.1**	**[28.5; 33.8]**	**--**
**Random effects model**			**31.6**	**[28.1; 35.1]**	**70.3%**
Heterogeneity: $I^2 = 37\%$, $\tau^2 = 0.0006$, $p = 0.18$					
**ET Status = Resistant**					
MONARCH-2	36	223	16.1	[11.6; 21.6]	14.8%
PALOMA-3	15	174	8.6	[4.9; 13.8]	15.0%
**Fixed effect model**		**397**	**11.8**	**[8.7; 15.0]**	**--**
**Random effects model**			**12.3**	**[4.9; 19.6]**	**29.7%**
Heterogeneity: $I^2 = 81\%$, $\tau^2 = 0.0023$, $p = 0.02$					
**Fixed effect model**		**1536**	**23.0**	**[21.0; 25.1]**	**--**
**Random effects model**			**26.4**	**[18.0; 34.8]**	**100.0%**
Heterogeneity: $I^2 = 94\%$, $\tau^2 = 0.0117$, $p < 0.01$					
Residual heterogeneity: $I^2 = 57\%$, $p = 0.04$					

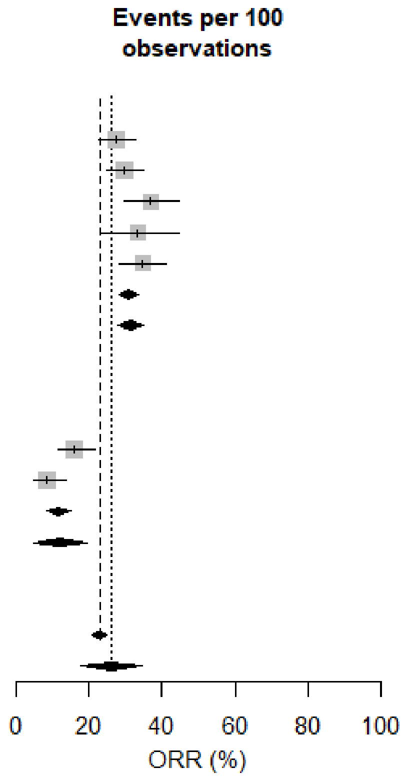

A meta-analysis of single proportions was carried out to obtain the pooled estimate of ORR in patients treated with endocrine therapy alone according their AI-sensititvity.

Figure S8. Bar-plot of pooled ORR in all randomly assigned patients according CDK 4/6 inhibitor

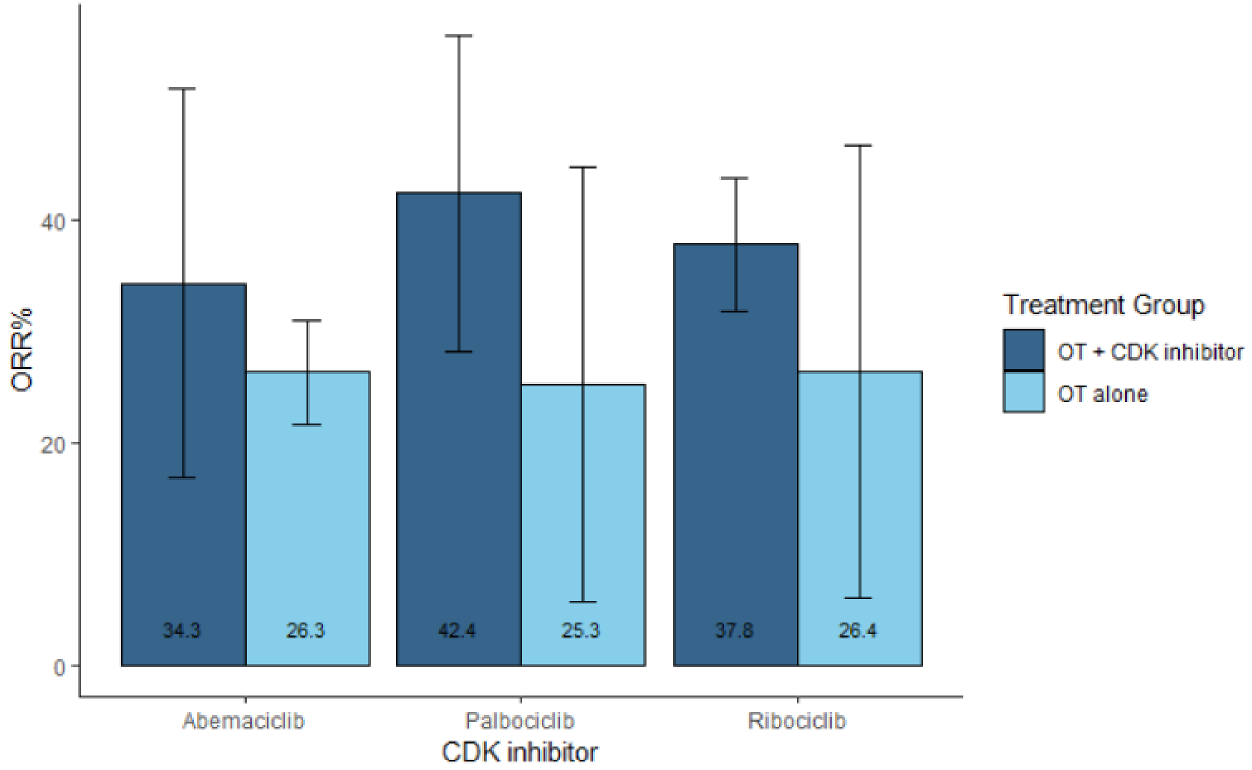

Abbreviations: OT, hormonal therapy (i.e. aromatase inhibitor, tamoxifene, fulvestrant); ORR, objective response rate.

Figure S9. Meta-analysis of overall survival (OS) in overall population

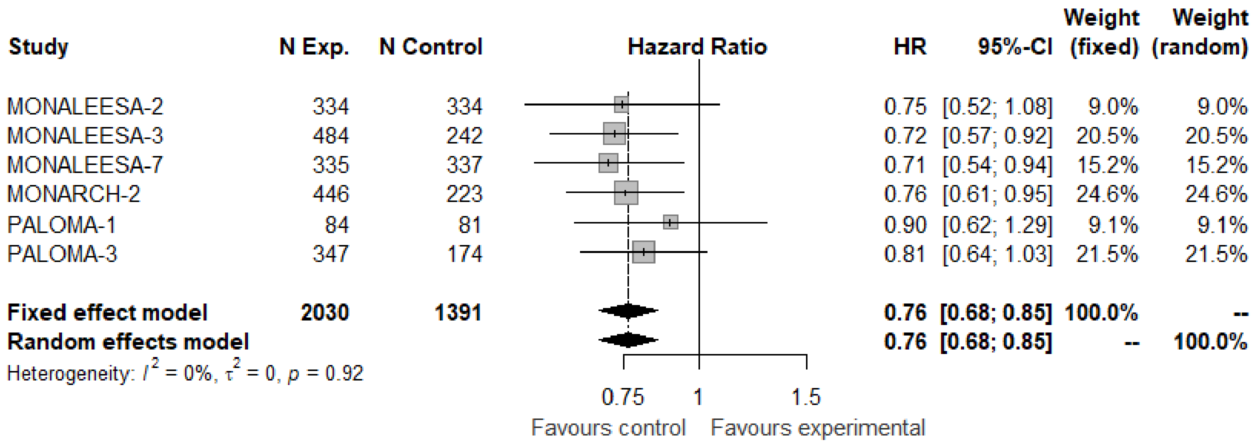

Table S3. PRISMA 2009 checklist

Section/topic	#	Checklist item	Reported on page #
**TITLE**			
Title	1	Identify the report as a systematic review, meta-analysis, or both.	1
**ABSTRACT**			
Structured summary	2	Provide a structured summary including, as applicable: background; objectives; data sources; study eligibility criteria, participants, and interventions; study appraisal and synthesis methods; results; limitations; conclusions and implications of key findings; systematic review registration number.	2
**INTRODUCTION**			
Rationale	3	Describe the rationale for the review in the context of what is already known.	3
Objectives	4	Provide an explicit statement of questions being addressed with reference to participants, interventions, comparisons, outcomes, and study design (PICOS).	5, 7
**METHODS**			
Protocol and registration	5	Indicate if a review protocol exists, if and where it can be accessed (e.g., Web address), and, if available, provide registration information including registration number.	6
Eligibility criteria	6	Specify study characteristics (e.g., PICOS, length of follow-up) and report characteristics (e.g., years considered, language, publication status) used as criteria for eligibility, giving rationale.	6
Information sources	7	Describe all information sources (e.g., databases with dates of coverage, contact with study authors to identify additional studies) in the search and date last searched.	6
Search	8	Present full electronic search strategy for at least one database, including any limits used, such that it could be repeated.	6, Table S1
Study selection	9	State the process for selecting studies (i.e., screening, eligibility, included in systematic review, and, if applicable, included in the meta-analysis).	7
Data collection process	10	Describe method of data extraction from reports (e.g., piloted forms, independently, in duplicate) and any processes for obtaining and confirming data from investigators.	7
Data items	11	List and define all variables for which data were sought (e.g., PICOS, funding sources) and any assumptions and simplifications made.	7
Risk of bias in individual studies	12	Describe methods used for assessing risk of bias of individual studies (including specification of whether this was done at the study or outcome level), and how this information is to be used in any data synthesis.	8
Summary measures	13	State the principal summary measures (e.g., risk ratio, difference in means).	8
Synthesis of results	14	Describe the methods of handling data and combining results of studies, if done, including measures of consistency (e.g., I^2) for each meta-analysis.	8

Section/topic	#	Checklist item	Reported on page #
Risk of bias across studies	15	Specify any assessment of risk of bias that may affect the cumulative evidence (e.g., publication bias, selective reporting within studies).	8
Additional analyses	16	Describe methods of additional analyses (e.g., sensitivity or subgroup analyses, meta-regression), if done, indicating which were pre-specified.	8
**RESULTS**			
Study selection	17	Give numbers of studies screened, assessed for eligibility, and included in the review, with reasons for exclusions at each stage, ideally with a flow diagram.	9-10
Study characteristics	18	For each study, present characteristics for which data were extracted (e.g., study size, PICOS, follow-up period) and provide the citations.	9-10
Risk of bias within studies	19	Present data on risk of bias of each study and, if available, any outcome level assessment (see item 12).	10
Results of individual studies	20	For all outcomes considered (benefits or harms), present, for each study: (a) simple summary data for each intervention group (b) effect estimates and confidence intervals, ideally with a forest plot.	Table 1
Synthesis of results	21	Present results of each meta-analysis done, including confidence intervals and measures of consistency.	10
Risk of bias across studies	22	Present results of any assessment of risk of bias across studies (see Item 15).	Fig-S2, Table S3
Additional analysis	23	Give results of additional analyses, if done (e.g., sensitivity or subgroup analyses, meta-regression [see Item 16]).	10-13, Fig. S4-S8
**DISCUSSION**			
Summary of evidence	24	Summarize the main findings including the strength of evidence for each main outcome; consider their relevance to key groups (e.g., healthcare providers, users, and policy makers).	14-16
Limitations	25	Discuss limitations at study and outcome level (e.g., risk of bias), and at review-level (e.g., incomplete retrieval of identified research, reporting bias).	14-16
Conclusions	26	Provide a general interpretation of the results in the context of other evidence, and implications for future research.	16
**FUNDING**			
Funding	27	Describe sources of funding for the systematic review and other support (e.g., supply of data); role of funders for the systematic review.	NA

From: Moher D, Liberati A, Tetzlaff J, Altman DG, The PRISMA Group (2009). Preferred Reporting Items for Systematic Reviews and Meta-Analyses: The PRISMA Statement. PLoS Med 6(7): e1000097. doi:10.1371/journal.pmed1000097

For more information, visit: **www.prisma-statement.org**.
